# Millimeter-wave pulsed heating *in vitro*: cell mortality and heat shock response

**DOI:** 10.1038/s41598-019-51731-7

**Published:** 2019-10-24

**Authors:** Rosa Orlacchio, Yann Le Page, Yves Le Dréan, Rémy Le Guével, Ronan Sauleau, Stanislav Alekseev, Maxim Zhadobov

**Affiliations:** 1 Univ Rennes, CNRS, IETR (Institut d’Electronique et de Télécommunication de Rennes) – UMR 6164, F-35000 Rennes, France; 2 Univ. Limoges, CNRS, XLIM, UMR 7252, F-87000 Limoges, France; 3 Univ Rennes, Inserm, EHESP, IRSET (Institut de Recherche en Santé Environnement et Travail) UMR_S 1085, F-35000 Rennes, France; 4 ImPACcell, SFR Biosit, Univ Rennes, Rennes, France; 5 Institute of Cell Biophysics of Russian Academy of Sciences, Institutskaia 3, 142290 Pushchino, Moscow Region Russia

**Keywords:** Biomedical engineering, Biological physics

## Abstract

Millimeter wave (MMW)-induced heating represents a promising alternative for non-invasive hyperthermia of superficial skin cancer, such as melanoma. Pulsed MMW-induced heating of tumors allows for reaching high peak temperatures without overheating surrounding tissues. Herein, for the first time, we evaluate apoptotic and heat shock responses of melanoma cells exposed *in vitro* to continuous (CW) or pulsed-wave (PW) amplitude-modulated MMW at 58.4 GHz with the same average temperature rise. Using an *ad hoc* exposure system, we generated 90 min pulse train with 1.5 s pulse duration, period of 20 s, amplitude of 10 °C, and steady-state temperature at the level of cells of 49.2 °C. The activation of Caspase-3 and phosphorylation of HSP27 were investigated using fluorescence microscopy to monitor the spatial variation of cellular response. Our results demonstrate that, under the considered exposure conditions, Caspase-3 activation was almost 5 times greater following PW exposure compared to CW. The relationship between the PW-induced cellular response and SAR-dependent temperature rise was non-linear. Phosphorylation of HSP27 was 58% stronger for PW compared to CW. It exhibits a plateau for the peak temperature ranging from 47.7 to 49.2 °C. Our results provide an insight into understanding of the cellular response to MMW-induced pulsed heating.

## Introduction

Most of the chemical reaction rates behind cellular processes are transient and temperature sensitive (empirical relationship is provided by the Arrhenius law). Depending on parameters and conditions of heating, two mechanisms are at the origin of cellular responses including i) inactivation of protein functions and enzymatic activity, and ii) activation of signaling pathways^1^. Protein and enzymatic inactivation is responsible for heat cytotoxicity^2,3^ and radio or chemo sensitization^4^ of the cells as responses to a severe heat shock (usually >43 °C), while induction of thermotolerance^5^ is the dominant activating response occurring when cells are exposed to sublethal temperatures, typically ranging from 39 to 42 °C.

It has been recently demonstrated that hyperthermic temperatures (i.e. 43 °C to 45 °C) are able to trigger both the extrinsic and intrinsic apoptotic pathways in melanoma cells^6^. In particular, it was shown that incubation of melanoma cells at 45 °C for 2 h induced activation of caspases-3,-6, and -7 up to 24 h (−3) and 72 h (−6, −7) post exposure. Caspases belong to a family of protease enzymes playing essential roles in programmed cell death; they are synthesized as inactive precursor molecules (pro-caspases) and are converted by proteolytic cleavage to active enzymes. The activation of caspases is also a marker of cellular damage in tissues.

Thermotolerance is due to existence of protein quality control response, which is one of the most conserved cytoprotective mechanisms in evolution^7^. In case of heat shock, cells overexpress chaperones and heat shock proteins (HSPs) that protect cellular proteins from misfolding and aggregation. HSPs, such as HSP27, have been identified as key determinants of cell survival because they also modulate apoptosis by directly interacting with components of the apoptotic machinery^8^. These proteins are the key factors in response to cellular stress and they are involved in many pathologies such as cancer or neurodegenerative diseases^9^. Their ability to bind to client proteins depends on their level of phosphorylation induced by heat shock response.

It was demonstrated that pulsed electromagnetically-induced heating can lead to stronger damage in cells compared to continuous heating^10^, allowing, in the case of thermo-oncological therapies, to decrease the treatment duration, reducing patient discomfort, and to eliminate or reduce the influence of blood perfusion as well as thermotolerance^11,12^. However, most of the studies dealing with pulsed heating have been performed to analyze the thermal damage threshold mainly in relation to ablation at temperatures exceeding the lethal threshold^13,14^. As the interest to pulsed thermal treatment of tumors is increasing, the study of the cellular response both in terms of heat induced damage and activation of cellular repair processes mediated by HSP induction is of importance.

Microwave (MW) frequencies have been exploited for invasive and non-invasive thermal treatments, mainly in the Industrial Scientific Medical (ISM) bands around 434 MHz, 915 MHz, 2.45 GHz, and 5.8 GHz^15–17^. The feasibility of using MW up to 18 GHz (e.g. 9.2 GHz^18^, 10 GHz^19^, 14.5 GHz^20^, and 18 GHz^21^) for tissue ablation with minimized invasiveness and collateral damages has been discussed in several studies. Furthermore, it has been recently demonstrated that the 20–100 GHz range can be employed for spatially-accurate focusing of heat inside the skin by varying frequency and exposure beam size, as well as by enforcing air convection to reduce overheating of skin surface shifting the maximum heating towards deeper skin layers^22^. The results suggested that the lower part of the millimeter-wave (MMW) range is an attractive alternative for non-invasive thermal treatment of skin cancer such as spreading melanoma.

The main objective of this study is to compare the responses of malignant melanoma cells to continuous and pulse-modulated MMW-induced heating. First, heat pulses were locally generated at the cellular level *in vitro* using an *ad hoc* MMW exposure system. Second, Caspase-3 (Casp-3) cleaved activation was evaluated in order to detect the effective heat damage in cells for the continuous and pulsed heating with the same average temperature rise. Third, the heat shock response was quantified by following the phosphorylation of HSP27. The fluorescence microscopy image analysis was used to analyze the cellular responses.

## Materials and Methods

### Exposure setup and electromagnetic dosimetry

**Figure 1:**
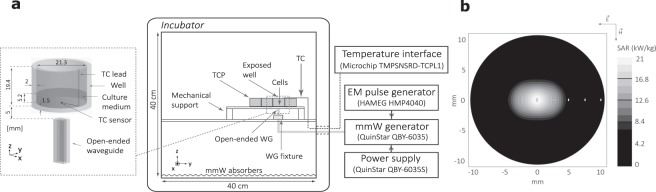
(**a**) Outline of the exposure setup. Cells located at the bottom of a well of a 12-well TCP were exposed by an open-ended WG inside the incubator at 32 °C (center). CAD model of the antenna and exposed well were used for computing SAR (left). Continuous wave and pulsed signals were generated at 58.4 GHz by a customized MMW generator controlled by an electromagnetic pulse generator. The temperature was monitored using a TC through a dedicated interface (right). (**b**) Computed SAR in the cell monolayer normalized to the antenna input power of 1W. White ellipses indicate the locations of TC sensors in temperature measurements.

Cells cultured in a standard 12-well tissue culture plate (TCP in Fig. 1a) made of polystyrene (353072, Microtest 96, Becton Dickinson, Franklin Lakes, NJ) were exposed from the bottom by an open-ended rectangular WR15 waveguide (WG) antenna (aperture size 3.81 × 1.905 mm^2^) located 5 mm from the plate inside a MEMMERT UNE400 incubator (Memmert, Schwabach, Germany) (Fig. 1a). A cell monolayer was located at the bottom of the well and covered by 2 ml of the culture medium. The antenna was fed by a set of standard V-band WG. Customized high-power generator (QuinStar Technology, Torrance, CA) operating at 58.4 GHz with an output power up to 3.7 W was used as a narrowband source in continuous-wave (CW) or pulsed-wave (PW) amplitude modulation regimes. Programmable pulse generator HMP 4040 (Hameg Instruments, Hampshire, UK) provided control voltage and current enabling amplitude modulation of the MMW radiation. The input power of the open-ended WG was systematically measured before experiments using V-band Agilent V8486A power meter (Agilent Technologies, Santa Clara, CA). To avoid the overheating of cells and compensate for a rapid temperature rise during the first minutes of exposure, the temperature of the incubator was set to 32 °C to obtain during the CW and PW exposures the desired average steady-state temperature of 42.3 °C, with the maximum peak temperature of PW exposure about 49 °C. To compute the electromagnetic power loss inside the well we used the numerical model illustrated in Fig. 1a (left). Only the antenna and one well of the TCP were simulated to reduce the computational volume represented for each simulation by about 30 million mesh cells. As power absorption within the exposed well is local and the specific absorption rate (SAR) is mainly concentrated at the bottom of the culture medium close to the well axis, the contribution of reflections from the neighboring empty wells to SAR distribution is negligible. As demonstrated in^23^, the effect of a thin monolayer (with a thickness of the order of several µm) on the absorbed power and resulting heating is negligible (less than 1%). Therefore the presence of a cell monolayer was neglected in simulations.

Electromagnetic properties of materials considered in modeling are given in Table 1 at 58.4 GHz. Complex permittivity of polystyrene was determined using a free-space technique with a transmission/reflection quasi-optical setup and ABmm millimeter-wave vector network analyzer^24^. Electromagnetic properties of distilled water and culture medium were measured using an open-ended coaxial probe DAK-1.2E (SPEAG, Zurich, Switzerland) and were found to be the same at the considered frequency within the measurement uncertainty (roughly ± 5%). Therefore the complex permittivity of the culture medium at higher temperatures was extrapolated using the model proposed by Ellison^25^. In electromagnetic computations, the permittivity, conductivity, and mass density were considered as temperature independent. As demonstrated in^23^, variations of these parameters due to the temperature increase by 10 °C result in the maximum change of peak SAR by only 1.5%.

**Table 1:** Relative permittivity and electrical conductivity of materials used in simulations.

Materials	ε	σ (S/m)
Background (air)	1	0
Antenna (perfect electric conductor)	—	∞
Tissue culture plate (polystyrene)	2.56	0.008
Distilled water at 37 °C	16.86	80.76

SAR at the bottom of the culture medium was computed using the finite-difference time-domain (FDTD) method. Electromagnetic simulations were carried out using the FDTD solver implemented in SEMCAD X (SPEAG, Zurich, Switzerland). Non-uniform adaptive three dimensional meshing was used to carefully account for elevated SAR gradients close to the bottom of the exposed liquid. All simulations were performed using a mesh with a cell size ranging from 5 µm (in liquid) up to 250 µm (in free space). Maximum grading ratio was set to 1.2. Perfectly matched layers (PML) absorbing boundary conditions were used. Minimal distance between the culture plate and boundaries was set to λ_0_/4. Note that the thermocouple (TC) was not included in simulations as its effect on heating is negligible^26,27^. Figure 1b shows SAR distribution in the cell monolayer. It was retrieved based on SAR computed in the culture medium by applying a correction factor extracted from^23^.

### Local temperature monitoring

Electromagnetic power dissipated in the cells and culture medium resulted in heating. Temperature was measured using a K type TC probe with the leads diameter of 75 µm (RS Components, Corby, UK). To record temperature we used Thermocouple Reference design (Microchip Technology, Chandler, AZ) with a sampling interval of 156 ms. As shown in Figure 1a, the tip of the TC was aligned with the exposure beam axis with its leads lying on the bottom of the well perpendicular to the *E*- plane. Such an orientation of TC prevents induction of currents in TC and related possible artefacts^26–32^. Temperature measurements were performed in separate experiments (culture medium only, without cells) in order to exclude any possible contamination as well as cell damage due to the presence of the TC. Each measurement was repeated 3 times.

### Cell culture and exposure protocol

The human malignant melanoma A375 cells were purchased from American Type Culture Collection (ATCC, Molsheim, France). These cells were cultured in Dulbecco’s modified Eagle medium (Gibco/Life Technologies, Carlsbad, CA) supplemented with 8% fetal calf serum (FCS), 1% antibiotics, and 1% L-glutamine, in a humidified incubator at 37 °C and 5% CO_2_. The medium was renewed every 2 days and the cells that reached 70 to 80% confluence were grown as monolayer cultures or passaged. To avoid any problem of senescence or drift of the cellular population, the experiments were conducted at earlier passages (between 4 and 10). For exposure, cells were seeded in 3 wells of 12-well tissue culture plate at a density of 30 000 cells per well. One well was exposed, one well was used as a negative control, and the third well was used as a positive control, which served as technical verification for detection of apoptosis or heat shock response (data not shown). Cells were treated with 100 µM of Antimycin A (Sigma-Aldrich, Saint-Quentin Fallavier, France) or with 5 µM of MG132 (Sigma-Aldrich) to detect Casp-3 activation or HSP27 phosphorylation, respectively. Two days after plating, the medium was replaced by a medium without sodium bicarbonate containing 4.6 mM of Hepes (Thermo Fisher Scientific, Waltham, MA) to maintain constant pH in the non-gassed incubator^33^. Then, the plate with cells was transferred to a standard incubator with exposure facilities. Before exposure, cells were incubated for 1 h at 32 °C. Heat shock response may appear after a certain delay following the exposure^34^, therefore cells were kept at 37 °C for 6 hours after exposure. Then, cells were fixed before proceeding to immunochemistry. Sham exposures were performed under identical experimental conditions, but with the generator switched off.

### Immunocytochemistry (ICC) and fluorescence analysis

The ICC and fluorescence analysis protocol was described in detail by Haas *et al*.^35^. Briefly, 6 hours after exposure, cells were fixed with 4% paraformaldehyde for 20 min at room temperature, washed twice with phosphate-buffered saline (PBS), and permeabilized for 10 min with 0.25% Triton X-100 in PBS. Unspecific binding of antibodies was blocked by incubating cells during 20 min in 1% bovine serum albumin (BSA, MP Biomedicals, Santa Ana, CA), 0.1% gelatin from cold water fish skin (Sigma-Aldrich, St Louis, MO), and 0.1% Triton X-100 in PBS. Cells were then incubated overnight, at 4 °C, with primary antibodies at 1:500 and 1:200 dilutions, respectively for cleaved Casp-3 (Cleaved Caspase-3 (Asp175), ref 96645, Cell Signaling Technology, Danvers, MA) and phosphorylated HSP27 (Phospho-HSP27 (Ser82), ref 2406, Cell Signaling Technology, Danvers, MA). After three successive washes using PBS supplemented with 0.1% Tween 20, cells were incubated 1 hour with secondary antibodies at 1:1000 dilution and Hoechst 33342 (10 µg/mL, Sigma-Aldrich) for nuclei counterstaining. Pictures of cells were taken and fluorescence of each cell was quantified using a Cellomics ArrayScan VTI HCS Reader (Thermo Fisher Scientific) at ImPACcell technological platform (Biosit, University of Rennes 1, Rennes, France). For each well, 121 pictures were taken following a square spiral from the center of the well covering a 30.25 mm² area (i.e. each picture had a 0.5 mm side and 0.25 mm² area). Distance equal to 0 mm was assigned to the picture taken in the center of the well aligned to the open-ended WG axis. All the cells in this picture were considered located at 0 mm from the center. Then, the distance from the center was calculated for each picture. Each picture contained an average number of about 100 cells allowing consistent average of the fluorescence value of the cells at each distance considered in the analysis.

### Calculation of the cumulative equivalent minutes (CEM43 °C)

The cumulative equivalent minutes at 43 °C (CEM43 °C) were calculated using the following formula^36^:

1 $$CEM{43}^{\circ }C=\mathop{\sum }\limits_{i}^{n}{t}_{i}{R}^{(43-\overline{T})}$$

where *n* represents the number of intervals in which the duration of the exposure has been derived, *t*_*i*_ is the *i*-th interval over which temperature is averaged (in minutes), $\overline{T}$ is the average temperature during the time interval *t*_*i*_, 43 °C is the reference temperature, and *R* is related to the temperature dependence of the rate of cell death. The parameter *R* used in this study is taken from^37^, derived from human skin cells *in vitro:*

2 $$R=\{\begin{array}{lll}0.233,\,T &  <  & {43}^{\circ }C\\ 0.428,\,T &  >  & {43}^{\circ }C\end{array}$$

In order to take into account the fast temperature variations during the pulse exposure, the averaging interval was set to 0.3 s.

### Statistical analysis

Three (n = 3) tissue culture plates were exposed or sham-exposed for each experimental condition. Statistical analyses were performed using SigmaPlot Statistics. The non-parametric Mann-Whitney Rank Sum test was used for the statistical comparisons of the data. For all tests, a p value < 0.05 was considered statistically significant. Results are presented as mean ± SEM (Standard Error of the Mean).

## Results

### Electromagnetic and thermal waveforms

**Figure 2:**
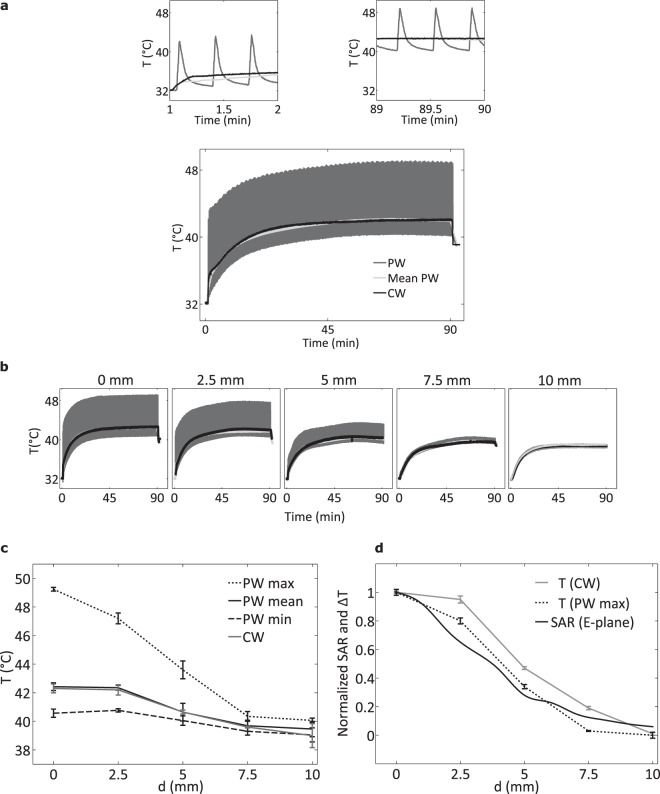
Temperature measured at the bottom of the exposed well. (**a**) Temperature dynamics in the center of the well bottom for PW and CW exposures. The subplots on the top illustrate the zoom for 1–2 min and 89–90 min intervals. (**b**) Temperature dynamics at different distances from the center of the well bottom in the lateral direction for 90 min of exposure (the locations of TC sensors are schematically illustrated in Fig. 1b). (**c**) Corresponding steady-state temperature rise after 90 min of exposure. (**d**) Normalized SAR along the E-plane and temperature rise ΔT (defined as the difference between the steady-state temperature at considered location and at 10 mm). Error bars indicate the SEM for three independent measurements.

Cells were exposed *in vitro* to CW or PW electromagnetic field at 58.4 GHz. Square-wave amplitude modulation was used to create electromagnetic pulses with the duration of 1.5 s. The peak power of PW exposure at the open-ended waveguide input was set to 3.7 W to generate thermal pulses with a peak amplitude of 10 °C in the center of the well (Fig. 2a). This power corresponds to a SAR level of 73.6 kW/kg in the cell monolayer and a temperature rise rate of 6.7 °C/s. The period of 20 s was selected to maintain the average PW heating (mean PW in Fig. 2a) below 43 °C (42.3 ± 0.31 °C). The latter was set to minimize the activation of HSPs that can have a protective action and reduce cell killing. At these conditions, the peak steady-state temperatures reached during PW exposure (48–49 °C) were high enough to destroy melanoma cells while retaining the average temperature at 42.3 °C. The average temperature was calculated by using a moving average filter with 75 s span. The power of CW exposure was adjusted to generate the same heating as the average temperature rise during the PW heating. It was equal to 250 mW corresponding to 4.9 kW/kg in terms of SAR. The exposure duration was set to 90 min corresponding to 270 pulses. The cells were exposed at steady-state temperature (41.6 < T ≤ 42.3 °C) for about 1 hour. This represents a typical duration employed in moderate hyperthermic oncological therapies^38,39^. Figure 2b,c show temperature rise measured at the bottom of the well at different distances from the well axis along *y* (*E*-plane in Fig. 1b). The corresponding SAR for PW and CW exposures was equal to 47.1, 20.6, 8.8, 4.4 kW/kg, and 3.1, 1.4, 0.6, 0.3 kW/kg for 2.5, 5, 7.5, 10 mm, respectively. ΔT profile for the peak temperature induced by PW exposure is similar to that of SAR (Fig. 2d), the maximum relative deviation is 25% at d = 2.5 mm.

CEM43 °C calculated for CW exposure was 23.9 ± 9.7 min. CEM43 °C delivered by pulsed heating was 502.67 ± 128.42 min, about 21 times greater than that delivered by continuous heating with the same average temperature. Values, reported as mean ± standard deviation over three temperature measurements, are representative of the thermal dose in the center of the well bottom, corresponding to the highest temperature induced in the cell monolayer. The CEM43 °C value equal to 502.67 min can be reached at 45.5 °C at continuous heating.

### MMW-induced thermal pulses induce stronger apoptosis in malignant melanoma cells compared to continuous heating with the same average temperature rise

Several *in vitro* and *in vivo* studies identified apoptosis as the key event responsible for induction of the cell death in response to thermal stress^40,41^. Apoptosis can be measured by using specific cleaved Casp-3 antibody. The Casp-3 is an effector protease, involved in the initiation of the programmed cell death signaling, and its activation is a hallmark of apoptosis^42^. It was found that the best way to assess the relative apoptotic activity in high-content fluorescence microscopy analysis is to calculate the percentage of cells above a certain threshold of activate Casp-3 labeling^43^. Therefore, we quantified Casp-3 activation as an indicator of apoptotic cells.

**Figure 3:**
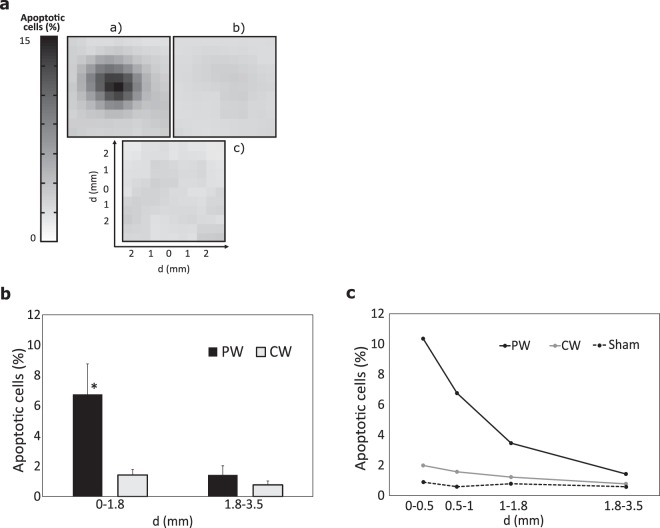
Percentage of apoptotic cells after PW and CW exposure with the same average temperature rise. (**a**) Spatial distribution of apoptotic cells for (**a**) PW, (**b**) CW, and (**c**) sham exposures. (**b**) Apoptotic response analyzed cell-by-cell 6 h post exposure shown as mean values (n = 3) ± SEM normalized to the sham. The data are averaged over the areas around the center of the well (i.e. 0–1.8 mm denotes the data averaged over the area with the radius of 1.8 mm, and 1.8–3.5 denotes the data averaged over 1.8 mm to 3.5 mm from the center). Asterisk (*) indicates statistical significance at p < 0.05. (**c**) The same data shown for the averaging with higher spatial resolution.

Figure 3a illustrates the distribution of apoptotic cells according to the detection of the Casp-3 activation following PW, CW, and sham exposures. It shows that the thermal pulses induce apoptosis, while CW heating with the same average temperature rise (Fig. 2a) does not induce any noticeable effect. Apoptosis triggering was almost 5 times greater for PW compared to CW in the area up to 1.8 mm from the well axis (Fig. 3b). For distances exceeding 1.8 mm (SAR, ΔT [PW max], and ΔT [CW] decrease by a factor of 1.3, 1.2, and 1.0 in regard to the peak values on the axis, respectively) this effect disappeared (apoptotic cells ratio decreased to 1.5%, and the difference between PW and CW inductions becomes statistically non-significant).

Interestingly, for PW, the decrease of apoptotic cells with distance from the well axis (Fig. 3c) was faster compared to the SAR and the thermal pulse amplitude drop (Fig. 2d). The percentage of apoptotic cells due to PW exposure was reduced by almost 7 times from 0 mm to 3.5 mm, while the SAR and the thermal pulse amplitude drop were only around 50%. This non-linear dependence of apoptotic cellular response on the thermal pulse amplitude may be explained by sensitivity of the melanoma cells to the properties of thermal pulses (e.g. duration, amplitude, period, temperature rise rate, and fast on/off temperature change) and/or by existence of a threshold in the capacity of cells to respond to thermal stress, such as limits in HSP regulation (see next sub-section). At the same time, the reduction of apoptotic cells by about 2.6 times from 0 mm to 3.5 mm for CW exposure was nearly proportional to the SAR and temperature drop, suggesting that the threshold was not reached.

These results suggest that, under the considered exposure conditions, PW exposure results in 5-fold stronger activation of Casp-3 compared to CW exposure with the same average heating.The relationship between the PW induced cellular response and SAR-dependent temperature rise is non-linear.

### MMW-induced thermal pulses induce stronger phosphorylation of HSP27 compared to continuous heating with the same average temperature rise

**Figure 4:**
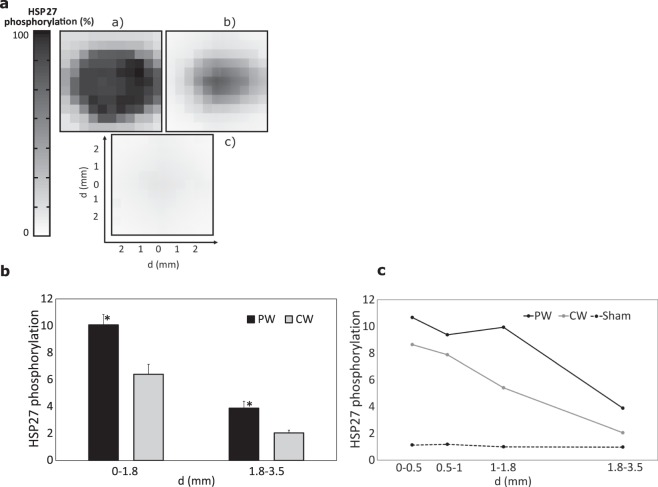
Phosphorylation of HSP27 after PW and CW exposure with the same average temperature rise. **(a)** Spatial distribution of the normalized intensity of the HSP27 phosphorylation for (**a**) PW, (**b**) CW, and (**c**) sham exposures. (**b**) Phosphorylation of HSP27 analyzed cell-by-cell 6 h post exposure shown as mean values (n = 3) ± SEM normalized to the sham. The data are averaged over the areas around the center of the well (i.e. 0–1.8 mm denotes the data averaged over the area with the radius of 1.8 mm, and 1.8–3.5 denotes the data averaged over 1.8 mm to 3.5 mm from the center). Asterisk (*) indicates statistical significance at p < 0.05. (**c**) The same data shown for the averaging with higher spatial resolution.

HSP expression in cells may correlate with healing or tissue damage. The small HSP27 can act as a molecular chaperone and protect cells against heat shock and oxidative stress when overexpressed^44^. In particular, physiological stimuli (such as redox signaling, cytokines, and growth factors) and various forms of stress (e.g. heat shock) dramatically increase the phosphorylation of HSP27^45^. In this study, HSP27 was used as a marker of heat-induced cellular stress. Figure 4a illustrates the distribution of the fluorescence intensity of cells following PW, CW, and sham exposures. Both PW and CW exposures induce phosphorylation of HSP27. The phosphorylation due to PW exposure was stronger than the one induced by CW at the same average temperature rise (Fig. 4b), i.e., 10.1 and 6.4-fold induction in respect to sham for PW and CW, respectively for d < 1.8 mm, and 4.3 and 2.2-fold induction for 1.8 mm < d < 3.5 mm.

For the PW exposure, in contrast to the activation of the apoptotic pathway, which rapidly decreases with distance from the well axis, the reduction of the phosphorylation of HSP27 with distance was much slower, i.e., it decays by a factor of 2.7 from 0 mm to 3.5 mm (Fig. 4c). It is interesting to note that the variation of phosphorylation of HSP27 in the cells exposed to PW has a plateau between 0 and 1.8 mm from the center (cellular response decreased only by 1.1 times), which is absent in the cells exposed to CW regime (Fig. 4c). This plateau probably corresponds to the phosphorylation of all HSP27 proteins present in cells. We have previously demonstrated that apoptosis drastically increases when this plateau is reached (Fig. 3c), which is consistent with the fact that refolding system is saturated. For the distances exceeding 1.8 mm, the phosphorylation of HSP27 was reduced by a factor of 2.6, similarly to the corresponding decrease of number apoptotic cells in the same region (factor 2.4). Although HSPs protect cells against lethal thermal injury, their induction and related thermotolerance indicate that significant injury has already occurred at cellular level^46^. Indeed, the highest level of phosphorylation of HSP27 induced by heat pulses (Fig. 4) is correlated to the strongest cellular death (Fig. 3), compared to cellular response after the exposure to CW. Our data suggest that when cells are exposed to temperature pulses reaching a maximum peak temperature exceeding 47.7 °C, they are not able to cope with the thermal stress fully activating repair processes, resulting in initiation of an apoptotic pathway mediated by Casp-3.

The reduction with distance of HSP27 phosphorylation induced by the CW exposure was steeper compared to the corresponding cellular apoptosis, i.e., heat shock response after CW exposure was reduced by about 4.2 times between 0 and 3.5 mm. The absence of the apoptotic response following CW heating may be related to the fact that cells exposed to continuous non-lethal temperatures <43 °C may initiate the cellular self-repair mechanism mediated by the induction of molecular chaperones (i.e. HSPs) preventing cells from protein denaturation and apoptosis^47^ (Fig. 3).

Our results suggest that, under the considered exposure conditions, both PW and CW exposures induce phosphorylation of HSP27. PW exposure results in 58% stronger phosphorylation of HSP27 compared to CW exposure with the same average heating. HSP27 phosphorylation induced by PW exhibits a plateau for the peak temperature ranging from 47.7 and 49.2 °C (the corresponding average temperature rise is between 42.2 and 42.4 °C).

## Discussion

Different parts of the microwave spectrum have been employed as a heating source in thermal therapies. Recently it has been demonstrated that the 20–100 GHz range can be employed for spatially-accurate focusing of heat inside skin by varying the frequency and exposure beam size, as well as by enforcing the air convection to reduce the overheating of skin surface shifting the maximum heating towards deeper skin layers^22^. Note that higher spatial resolution can be achieved in this band compared to lower MW frequencies and locally higher temperature elevations and gradients can be induced due to the increased transmission at the air/tissue interface and more localized absorption. This makes promising application of MMW for non-invasive thermal treatment of skin cancer such as spreading melanoma. Furthermore, in contrast to CW heating, which characterizes conventional hyperthermia, PW exposure may induce heat pulses with high peak temperatures confined to small areas of interest (typically from tenths of mm to several mm for MMW), eluding the injury of surrounding healthy tissues.

Induction of cell death as a response to hyperthermia in malignant melanoma cells was investigated in several studies. In particular, *in vitro* studies performed on melanoma cells have demonstrated a variety of effects for temperatures ranging from 41 to 48 °C including the reduction of cell viability in a time and temperature-dependent manner^48^, activation of an apoptotic pathway^6^ or the endoplasmic reticulum (ER) stress, and ER-mediated apoptosis^49^. However, the above-mentioned studies exploited the cytotoxic effect of heat delivered in a continuous manner. For the best of our knowledge, the hyperthermic response of melanoma cells, following the exposure to pulsed MMW-induced heating, has never been investigated so far.

In this study, we compared the spatial distribution of the cellular response *in vitro* following the exposure to CW and PW MMW-induced heating using an experimental approach based on fluorescence microscopy image analysis, analyzing cell-by-cell the cellular response. The reliability of our experimental protocol is confirmed by the very small baseline cellular apoptosis (<1%, Fig. 3) and phosphorylation of HSP27 (Fig. 4) that did not show any dependence on the distance from the well axis. Heat cytotoxicity was determined via activation of the Casp-3, which is a marker of the cell’s entry point into the apoptotic signaling pathway. The results were correlated with induction of the HSP27 phosphorylation. Different cellular responses for CW and PW MMW-induced heating with the same average temperature dynamics were analyzed in detail on A375 malignant melanoma cell line. The analysis of the effects of exposure to MMW-induced heating on different cell lines constitutes one of the perspectives of a future study.

The CW exposure results in a mild heat shock induction, which generates a thermotolerance response through HSP27 phosphorylation (Fig. 4), which in turn inhibits the activation of the Casp-3 (Fig. 3). This is in agreement with previous reports^50–54^ demonstrating that induction of HSP27 may interact with the apoptotic machinery, exercising anti-apoptotic activity, by following different paths. Numerous studies evidenced that HSP27 specifically interferes with the mitochondrial (i.e. intrinsic) pathway of Caspase-dependent cell death^34,55–57^. For example, Pandey *et al*.^34^, showed that HSP27 functions as an intracellular inhibitor of Casp-3 activation acting downstream to mitochondrial release of cytochrome *c*, Apaf-1, and Casp-9 activation. In addition, phosphorylated dimers of HSP27 may also inhibit an extrinsic pathway of cellular apoptosis^58^. Our study suggests that moderate temperature below 43 °C is not sufficient to induce the cellular death of malignant melanoma cells, thus being not suitable for the thermo-oncological treatment of superficial melanoma.

In contrast to the CW exposure, a train (90 min) of short (1.5 s) heat pulses induces cellular damage as shown by the activation of the Casp-3 (Fig. 3), in spite of the higher phosphorylation of HSP27 (Fig. 4). Several studies showed that small HSP exhibits temperature-dependent chaperone like-activity preventing the aggregation of stressed proteins^59^. In particular, it has been demonstrated by previous studies that HSP27 undergoes thermally induced self-association^60^, leading to increased oligomeric size, which correlates with increase in its chaperone-like activity^61^. For instance, Garolla and Mauk^61^ evaluated the chaperone activity of HSP27 as its ability to inhibit dithiothretoil-induced insulin aggregation as a function of the temperature, in the 20–48 °C temperature interval. Their results showed that HSP27 ability to protect cells increases with the temperature, exhibiting a sharp increase in the 34–43 °C range. At higher temperature, i.e., 43–48 °C, chaperone activity increases only slightly exhibiting an apparent plateau. These results are consistent with the outcomes of the present study. The analysis of the dependence of the phosphorylation of HSP27 after PW exposure on the distances from the center showed that, in the region 0–1.8 mm, the phosphorylation of HSP27 reached the maximal level exhibiting a plateau (Fig. 4c). This region corresponds to the peak temperature rise of 47.7–49.2 °C (Fig. 2b). These results suggest that the amplitude of thermal pulses plays an important role in inducing apoptosis in cells. Cells become more vulnerable to heat damage at peak temperatures within the 47–49 °C interval. At these temperatures, in spite of the maximum chaperon activation, the cell protection mechanism based on activation of HSP27 phosphorylation and other heat shock proteins does not cope with increasing heat damage of cells. Previous studies evidenced that, depending on the intensity of the stress, occurrence of phosphorylation induces the dissociation of large non-phosphorylated HSP27^44^. As long as significant amounts of large HSP27 oligomers could be formed, *in vitro* chaperone properties preventing thermal aggregation are detected. However, overexpression of phosphorylation of HSP27 down-regulates its chaperone activity by decreasing oligomerization of the protein and its consequent ability to trap and refold the thermally stressed proteins^44^. These observations are consistent with the results of our study, demonstrating that greater phosphorylation of HSP27, probably associated with lower oligomerization of the proteins, results in a decrease of its chaperone-like activity as evidenced by the increase of the number of cells undergoing apoptosis.

In order to quantify the thermal dose delivered to the exposed cells, we calculated the cumulative equivalent minutes (CEM43 °C). In our experiments, the thermal dose delivered by pulsed heating (502.7 min) was 21 times greater than that delivered by CW heating (23.9 min). To obtain the dose 502.7 min for CW exposure the steady-state temperature should be 45.5 °C. At pulsed heating, the temperatures in pulses are higher. The main contribution to the achievement of the high level of CEM43 °C during pulsed heating is related to temperatures in the interval between 47 and 49 °C. As shown in this study, these temperatures are more effective in inducing apoptosis. Nevertheless, we cannot exclude that CW exposure at the same dose (502.7 min; 45.5 °C) could induce a similar effect. A comparative study of the effects of PW and CW exposures at the same thermal dose on the cell damage is out of the scope of this study but constitutes one of its perspectives. In spite of the high thermal dose, heat pulses produce relatively low average temperature elevation. We hypothesize that this will prevent in tumors and surrounding tissues secondary long-lasting thermally-activated processes (for example, elevation of blood flow) as well as overheating of healthy tissues. We also hypothesize that this will make pulsed heating beneficial in application for inducing the cell damage.

A heat pulse is characterized not only by minimal, mean, and peak temperature but also by other parameters, such as the temperature rise rate. While not providing direct evidence in terms of cellular stress, several studies suggested that temperature rise could lead to changes at the cellular level. For instance, it was demonstrated that the transient heating (i.e. warm-up phase) up to several °C at 75 GHz leaded to changes in the membrane potential and consequently in the firing rate of neurons^12,62^. These changes were dependent on the temperature rise rate. Another group demonstrated that temperature increase at 10 °C/s rate caused a temporary cessation in the firing of the pacemaker neurons^63^. Furthermore, it was reported that the viability of liver cancer cells exposed at 100 MHz depends on the temperature rise rate^64^. In this case, the cell viability was unaffected by temperature rise rates below 10 °C/s and decreased to 90% for the temperature rise rate of 50 °C/s. Note that the effect was shown to be frequency dependent, and the cell mortality threshold shifted towards lower temperature rise rates when increasing the frequency to 2.45 GHz. The temperature rise rate of pulses used in our study is 6.7 °C/s, and we cannot exclude that the cell damage also depends on it. The effects of the amplitude of the pulse and temperature rise rate cannot be easily discriminated. With increasing the distance from the center of a well the amplitude of the thermal pulses decreases (Fig. 2) accompanied with decreasing the temperature rise rate. One of the possible ways to get an insight into the role of the temperature rise rate is to apply the temperature pulses of different duration and shape. This constitutes one of the perspectives of this work.

This study paves the way towards destruction of melanoma cells at low average heating by means of amplitude-modulated MMW. It demonstrates that the MMW-induced heat pulses, with duration of 1.5 s, induce cellular injury in the exposed cells, in contrast to continuous heating with the same average temperature rise. Optimization of the pulse shape, in particular in terms of its amplitude, rise time, duration, as well as duty cycle, represents a challenging problem for future studies in order to achieve the strongest cellular apoptosis in the minimally invasive way.

## Data Availability

All data generated or analysed during this study are available from the corresponding author on reasonable request.
